# Hypouricemia and Urate Transporters

**DOI:** 10.3390/biomedicines10030652

**Published:** 2022-03-11

**Authors:** Naoyuki Otani, Motoshi Ouchi, Kazuharu Misawa, Ichiro Hisatome, Naohiko Anzai

**Affiliations:** 1 Department of Clinical Pharmacology and Therapeutics, Faculty of Medicine, Oita University, Yufu 879-5593, Oita, Japan; naoyuki@oita-u.ac.jp; 2 Department of Pharmacology and Toxicology, Dokkyo Medical University School of Medicine, Mibu 321-0293, Tochigi, Japan; ouchi@dokkyomed.ac.jp; 3 Department of Human Genetics, Yokohama City University Graduate School of Medicine, Yokohama 236-0004, Kanagawa, Japan; kazu_misawa@hotmail.com; 4 Yonago Medical Center, National Hospital Organization, Yonago 683-0006, Tottori, Japan; hisatome@tottori-u.ac.jp; 5 Department of Genetic Medicine and Regenerative Therapeutics, Institute of Regenerative Medicine and Biofunction, Graduate School of Medical Sciences, Tottori University, Yonago 680-8550, Tottori, Japan; 6 Department of Pharmacology, Chiba University Graduate School of Medicine, Chiba 260-8670, Chiba, Japan

**Keywords:** hypouricemia, renal hypouricemia, urate transporter, URAT1, xanthinuria

## Abstract

Hypouricemia is recognized as a rare disorder, defined as a serum uric acid level of 2.0 mg/dL or less. Hypouricemia is divided into an overexcretion type and an underproduction type. The former typical disease is xanthinuria, and the latter is renal hypouricemia (RHUC). The frequency of nephrogenic hypouricemia due to a deficiency of URAT1 is high in Japan, accounting for most asymptomatic and persistent cases of hypouricemia. RHUC results in a high risk of exercise-induced acute kidney injury and urolithiasis. It is vital to promote research on RHUC, as this will lead not only to the elucidation of its pathophysiology but also to the development of new treatments for gout and hyperuricemia.

## 1. Introduction

The serum uric acid level depends on the balance between the production and excretion of uric acid. Healthy people maintain serum uric acid levels through the balance of uric acid production and excretion; imbalances can increase or decrease uric acid levels, causing hyperuricemia and hypouricemia. Hyperuricemia is defined by the solubility of uric acid when the serum uric acid level exceeds 7.0 mg/dL, regardless of gender [1], and uric acid accumulates in the kidneys and joints. In recent years, some epidemiological studies have suggested that it may cause the progression of renal disorders [2]. Several disorders are associated with serum uric acid level changes, and hyperuricemia is associated with common disorders such as hypertension, gout, cardiovascular disease, and chronic kidney disease (CKD) [3]. In contrast, hypouricemia was originally considered a biochemical disorder of no clinical significance, and the absence of symptoms may explain the lack of awareness of hypouricemia among healthcare providers. However, several studies have shown that patients with renal hypouricemia (RHUC) are at increased risk of exercise-induced acute kidney injury (EIAKI) and urolithiasis. In addition, these patients may develop chronic kidney disease [4,5,6,7]. Thus, hypouricemia has gradually been recognized as a pathological condition.

In recent years, studies of hypouricemia have provided new insights into the transport mechanism and have clarified the physiological function of uric acid transport. Uric acid transporters play an essential role in uric acid transport, and genes and transporters that correlate with serum uric acid levels have been identified. Urate transporter 1 (URAT1) and glucose transporter 9 (GLUT9) are transporters involved in uric acid reabsorption in renal proximal tubules. Renal hypouricemia is classified as type 1 when caused by abnormalities in the *URAT1* gene and type 2 when caused by abnormalities in the *GLUT9*/*URATv1* gene. In addition to known inborn errors and abnormalities of purine metabolism in hereditary renal diseases, the pathogenesis of uric acid metabolism in hereditary renal diseases has been attributed to abnormalities in the transporter genes. Here, we discuss hypouricemia from the perspective of uric acid transporters.

## 2. Molecular Mechanism of Urate in the Body

The production and metabolism of uric acid is a process involving a variety of factors that regulate hepatic production and renal and intestinal excretion. Exogenous production varies considerably with diet, whereas endogenous production is derived primarily from the liver and other tissues, including muscles and the vascular endothelium [8,9]. Inosine produced from adenosine is converted to the purine base hypoxanthine by purine nucleoside phosphorylase (PNP). Hypoxanthine is oxidized to form xanthine by xanthine dehydrogenase (XDH)/xanthine oxidoreductase (XOR). Xanthine is oxidized by XDH/XOR to form the final product, uric acid. At physiological pH, uric acid is a weak acid with a pKa of 5.8. Uric acid exists primarily as urate, the salt of uric acid. The concentration of serum uric acid increases in tandem with uric acid crystal formation. The solubility of uric acid in water is low, and in humans, the average concentration of serum uric acid is close to the solubility limit (6.8 mg/dL). Uric acid levels higher than 6.8 mg/dL cause uric acid crystals to form as monosodium urate [10]. In other mammals, uric acid is further degraded by uricase, an enzyme that primates lack, to produce allantoin [11], which is more water-soluble than uric acid and is efficiently excreted in urine [12].

In general, two-thirds of urate is excreted in the urine from the kidneys via the “renal excretion” pathway, whereas the remaining one-third is excreted via the “extra-renal excretion” pathway, such as intestinal excretion [13]. Urate is filtered by the glomeruli and reabsorbed by the proximal tubule with a normal fractional excretion of 10% [9,14]. This reabsorption is primarily accomplished at the proximal tubular by transporters that exchange intracellular anions for urate. Three urate transporters—URAT1/*SLC22A12* [15], GLUT9/*SLC2A9* [16,17], and ATP-binding cassette subfamily G member 2 (ABCG2)/breast cancer resistance protein (BCRP) [18,19]—are located in the proximal tubule and play important roles in the regulation of serum uric acid. Urate transporter disorders are caused by the dysfunction of these transporters. URAT1/*SLC22A12* is a typical urate reabsorption transporter expressed in proximal tubular cells. URAT1, encoded by *SLC22A12*, has 12 transmembrane domains and belongs to the organic anion transporter (OAT) family [15,20]. URAT1 transports urate in a time- and concentration-dependent manner, and its *K*_m_ value of approximately 370 μM is close to the value of serum urate in healthy humans (250–400 μM). URAT1 is a molecular target for uricosuric agents such as benzbromarone, probenecid, losartan, irbesartan [21], and dotinurad [22,23]. These drugs inhibit the reabsorption of urate in the lumen of the proximal tubule [15,24]. Loss-of-function mutations of URAT1/*SLC22A12* cause RHUC. The serum uric acid levels of patients with RHUC are very low, sometimes typically ≤ 2.0 mg/dL. This indicates the contribution of normal URAT1 in maintaining serum uric acid levels. The glucose transporter family gene *SLC2A9* encodes GLUT9, and *SLC2A9* expression levels are related to serum uric acid levels [25]. Anzai et al. [17] analyzed GLUT9 in a Xenopus oocyte expression system and first reported that GLUT9 is a voltage-driven urate efflux transporter. GLUT9 is located in the basolateral membrane of proximal tubule [26]. Urate taken up into proximal tubules is released on the vascular side by GLUT9. Mutations in GLUT9 have been reported to cause severe hypouricemia [16]. ABCG2/BCRP is an ATP-binding cassette (ABC) transporter encoded by the *ABCG2* gene. The transporters are localized on the luminal side of small intestinal epithelial cells and in renal proximal tubules and are responsible for urate excretion in the stool and urine, respectively [27]. Hyperuricemia associated with decreased function of ABCG2 causes reduced excretion of uric acid from tissues other than the kidney (intestinal tract) and is physiologically important in excretion of uric acid [11]. Other transporters related to uric acid have been reported. Transport of urate through the vascular side membrane to the proximal tubular cells is mediated by OAT1 or OAT3. Lesinurad (RDEA594), a novel uric acid-lowering agent, is an orally administered agent that selectively inhibits URAT1 and OAT4 in kidneys. It thereby inhibits uric acid reabsorption, resulting in increased renal uric acid excretion and decreased serum uric acid levels [28]. Transport across the lumen side membrane to proximal tubular cells for secretion into urine is facilitated by the sodium-dependent phosphate transporter type 4 (NPT4) encoded by the *SLC17A3* gene [20,29]. Monocarboxylate transporter 9 (MCT9), encoded by the *SLC16A9* gene, is a bidirectional urate transporter, presumably involved in the vascular side efflux of urate, but the functional mechanism of MCT9 remains unresolved [20,30].

## 3. Definition and Prevalence of Hypouricemia

**Figure 1:**
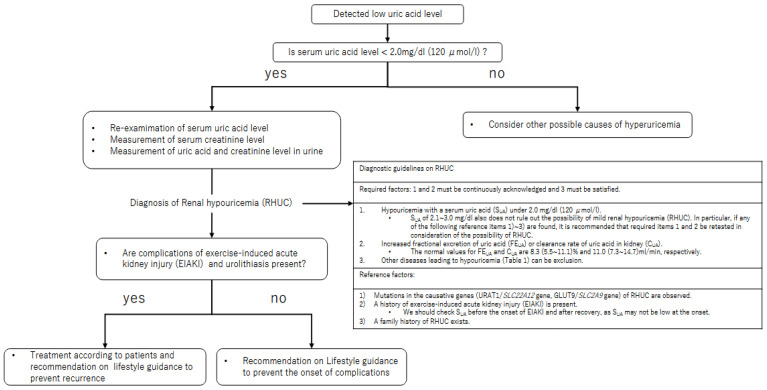
Flowchart for diagnosing RHUC in routine clinical practice. When low serum uric acid levels are detected, especially when the value is <2.0 mg/dL, uric acid and creatinine levels in blood and urine should be retested, and a diagnosis should be made based on the diagnostic guidelines on renal hypouricemia (RHUC). Modified from Nakayama et al. [34] under the terms of the Creative Commons Attribution 4.0 International License (http://creativecommons.org/licenses/by/4.0/ access date: 4 May 2022).

Hypouricemia is a disease in which the serum uric acid level is low. Most patients with hypouricemia are asymptomatic and are identified by chance on medical examination. Although there has been no clear definition of hypouricemia, 2.0 mg/dL or less has generally been used as a reference value for hyperuricemia [31]. In a study by Wakasugi et al., that examined the prevalence of hypouricemia in Japanese using this criterion, it occurred in 193 of 90,710 men (0.2%) and 540 of 136,935 women (0.4%) [32]. Hisatome et al., measured serum uric acid in 3258 Japanese outpatients; 5 of them had persistent hypouricemia, and 4 of the 5 patients were proven to have RHUC [33]. In addition, although most of the hypouricemia was RHUC, the prevalence of hypouricemia and the prevalence of RHUC were presumed to be almost the same. Clinical practice guidelines for RHUC have recently been developed [34,35]. Based on this clinical algorithm (Figure 1), some medical examinations for RHUC are strongly recommended if patients have a serum uric acid level of 2.0 mg/dL or less. It is recommended to measure blood uric acid level multiple times, in addition to measuring urinary uric acid excretion and uric acid clearance or urinary uric acid excretion rate.

Hypouricemia is caused by an increase in the renal clearance of uric acid, as well as a decrease in the formation of uric acid [36]. Therefore, hypouricemia is divided into an overexcretion type, in which there is increased uric acid excretion, and an underproduction type, in which there is decreased uric acid production. There are several specific illnesses that cause hypouricemia. Hypouricemia, which involves increased uric acid excretion, includes, in addition to RHUC, Fanconi syndrome, Wilson’s disease, syndrome of inappropriate antidiuretic hormone secretion (SIADH), malignancy, diabetes, and drug-induced hypouricemia. Fanconi syndrome includes the dysfunction of the proximal tubules, causing hypouricemia [37]. Hypouricemia, which is a reduction in uric acid production, includes xanthinuria (type 1 and type 2), molybdenum cofactor deficiency, PNP deficiency, phosphoribosylpyrophosphate (PRPP) synthetase deficiency, severe liver disorder, undernutrition, and drug-induced hypouricemia (Table 1). A typical disease of the former is RHUC caused by URAT1 and GLUT9 deficiency, and the latter is xanthinuria, which is a xanthine dehydrogenase deficiency. The frequency of RHUC due to URAT1 deficiency is high, and it is an asymptomatic and persistent form of hypouricemia. Xanthinuria and purine nucleotide phosphorylase deficiency are also causes of very low serum uric acid, but these are very rare [38,39]. Renal hypouricemia and xanthinuria often present with a serum uric acid level of 1 mg/dL or less, and other diseases of hypouricemia also sometimes result in a serum uric acid level of 2.0 mg/dL or less.

Hypouricemia was first reported in 1950 [40], and Akaoka et al., first reported RHUC in 1975 [41]. RHUC is relatively more common in the Japanese population than in other countries (about 0.3%) [16,31,42]. RHUC has also been reported in Jewish [43] and Roma [44] populations. According to Ogino et al., serum uric acid was measured in 586 normal subjects (347 men; 239 women) and 1220 inpatients in Japan. Hypouricemia was found in 0.34% of the normal subjects (no men; two women) and 2.54% of the inpatients. Hypouricemia has been occasionally diagnosed in inpatients and appears to be primarily caused by medication, neoplastic diseases, liver disorders, and transfusions [45]. In the Japanese study by Wakasugi et al., it occurred in 193 of 90,710 men (0.2%) and 540 of 136,935 women (0.4%) [32]. According to a Korean study by Son et al., the prevalence was 4.14% (299/7223 subjects) among inpatients and 0.53% (125/23,534 subjects) in outpatients, with an overall prevalence of 1.39% (424/30,757 subjects) [46]. Gresser et al., reported four studies since 1962 on uric acid levels in blood donors from 3200 subjects (2097 men and 1103 women) in southern Germany (Bavaria). Hypouricemia, a uric acid level below 2.0 mg/dL, was observed in three women not receiving medication and zero men [47].

## 4. Risk of Developing Diseases Associated with Hypouricemia

Hypouricemia is associated with the risk of developing some diseases. Uric acid can lower the risk of neurodegenerative conditions such as Alzheimer’s and Parkinson’s diseases. A recent meta-analysis that included 46 papers (*n* = 16,688 participants) dealing with all causes of dementia and 22 papers dealing with Alzheimer’s disease diagnosis revealed that serum uric acid levels were lower in dementia and Alzheimer’s disease patients when compared with levels in healthy patients [48,49]. In a meta-analysis by Wen et al. [50], which investigated 4646 participants (2379 Parkinson’s disease patients and 2267 controls) from 13 studies, Parkinson’s disease patients had lower serum uric acid levels than control patients, with no significant differences in age, sex, and geographic providence. These results are also related to the antioxidant effects of uric acid [51]. Therefore, the latest European Alliance of Associations for Rheumatology (EULAR) recommendations for the management of gout recommend that individuals maintain serum uric acid levels of <3 mg/dL for an extended period of time [52].

D’Silva et al. [53] reported ~30% higher all-cause mortality risk among US men with a serum uric acid level of <4 mg/dL, which was also associated with a nearly 3-fold higher risk of diabetes mellitus-related mortality in a US national survey; however, they found no long-term excess mortality risk in women with uric acid levels as low as <3 mg/dL. Serum uric acid levels were higher in prediabetics than in non-diabetics, and the uric acid level decreased with increasing duration of diabetes [54]. Prediabetes and obesity are associated with rising serum uric acid levels because of insulin resistance, and long-term diabetes mellitus is associated with lower serum uric acid levels and uricosuria because of impaired urate reabsorption by proximal tubules of the kidney. An EPOCH-JAPAN study revealed a J- or U-shaped relationship between serum uric acid levels and cardiovascular mortality. Hypouricemia in this study was driven by cardiovascular disease-related mortality [55]. Moreover, Kuwabara et al. [56] showed that women with serum uric acid levels of <2 mg/dL without cardiometabolic disease at baseline were more likely to develop incident chronic kidney disease and hypertension. In Korea, hypouricemia in men was reported to be driven by malignancy-related mortality in addition to cardiovascular disease-related mortality [57]. Furthermore, low serum uric acid levels are associated with malnutrition. In Taiwan, an analysis of data from participants 65 years or older concluded that a higher risk of mortality from hypouricemia was due to malnutrition as determined by BMI and serum albumin levels [58].

## 5. Uric Acid Levels Are Associated with Diabetes Mellitus

Hyperuricemia is closely linked to diabetes. Some reports described that insulin resistance correlates with serum uric acid levels and inversely correlates with the renal clearance of uric acid [59,60]. In addition, insulin has been suggested to cause antiuricosuria in the renal tubule [60]. The serum uric acid level has been shown to decrease as diabetes progresses [54,61,62,63]. One reason for such a finding is that the renal uric acid excretion rate is probably dependent on the degree of glycosuria [62]. Under conditions of poor glycemic control, we often meet patients with normal uric acid levels. Those patients may have worsening uric acid levels as their glycemic control improves. This condition may occur because the low insulin level decreases the reabsorption of uric acid in the proximal tubules. In contrast, the osmotic diuresis observed in hyperglycemia is presumably related to the urinary excretion of uric acid.

Sodium/glucose co-transporter-2 (SGLT2) inhibitors, which many diabetes patients use, lower blood uric acid levels by increasing urinary glucose and increasing the excretion of uric acid, which is co-transported with glucose [64,65,66]. Chino et al. [67] reported a decrease in the serum uric acid level and an increase in the urinary excretion rate of uric acid when luseogliflozin was administered to healthy subjects. The effects were postulated to be caused by osmotic diuresis and effect uric acid handling in kidneys. However, the mechanisms underlying the uricosuric effects remain unclear. Additionally, a reduction in serum uric acid has been reported in patients with familial renal glycosuria associated with mutations in *SGLT2* [68]. In a meta-analysis, Zhao et al. [69] examined the effects of empagliflozin on blood pressure, glucose, lipids, uric acid, estimated glomerular filtration rate, and body weight in patients with type 2 diabetes mellitus. In that study, empagliflozin at 10 or 25 mg reduced uric acid levels. The uric acid results in that meta-analysis were consistent with the results of the EMPA-REG OUTCOME trial [70], suggesting that empagliflozin had beneficial effects on these cardiovascular risk factors in patients with type 2 diabetes mellitus. Additionally, empagliflozin also reduced systolic and diastolic blood pressures, hemoglobin A1c, fasting blood glucose, and body weight in patients with type 2 diabetes mellitus. Uric acid is also an important issue in clinical diabetes, metabolism, and nutrition. The relationship between transporters and uric acid is very important. Further investigation should be conducted on the relationship between glucose and uric acid and the relationship with transporters, including URAT1/*SLC22A12* and GLUT9/*SLC2A9*.

## 6. Renal Hypouricemia (RHUC)

**Figure 2:**
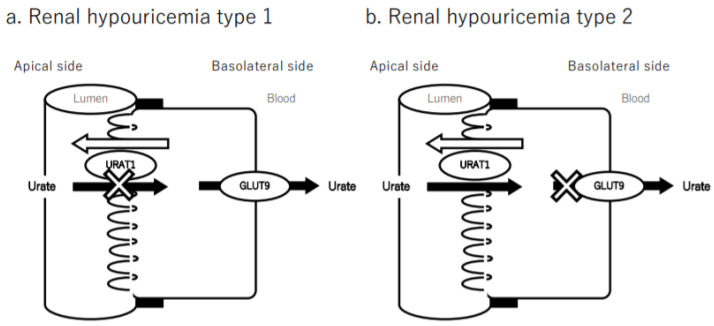
Transport mechanism of urate in the kidney. Urate is freely filtered at the glomerulus; most of it is reabsorbed via the urate transporter in the proximal tubule, while about one-tenth is secreted into the proximal tubules. URAT1/*SLC22A12* reabsorbs urate and is located in the apical membrane of the proximal tubules, and GLUT9/*SLC2A9* excretes urate through the basolateral side of the proximal tubule cells. The loss of function of these transporters due to genetic mutations causes RHUC, which is referred to as RHUC type 1 due to URAT1/*SLC22A12* (**a**) and RHUC type 2 due to GLUT9/*SLC2A9* (**b**).

Genetic studies have identified defects in the URAT1/SLC22A12 and GLUT9/*SLC2A9* urate transporter genes as causes of RHUC type 1 and type 2 [15,38,39], respectively (Figure 2). RHUC type 1 is caused by loss-of-function mutations. For the *SLC22A12* gene, which is mapped to chromosome 11q13 and encodes the urate-anion exchanger URAT1 [15], RHUC type 2 is caused by an inactivating mutation in the *SLC2A9* gene that encodes GLUT9, which maps to chromosome 4p15.3-p16 [16].

URAT1, non-functional variants of R90H (rs121907896), and W258X (rs121907892) are mutations that are frequent causes of RHUC in the Japanese population [38,39,71,72,73]. According to Sakiyama et al., the URAT1 non-functional alleles of R90H and W258X were found to significantly reduce serum urate in a study of 4902 healthcare participants. Men with one or two non-functional alleles of URAT1 showed a marked reduction in serum uric acid of 2.19 or 5.42 mg/dL, respectively. Similarly, women with one or two non-functional alleles of URAT1 also showed reduced serum uric acid concentrations of 1.08 or 3.89 mg/dL, respectively [71]. Moreover, they revealed sex-dependent effects on serum uric acid of URAT1 non-functional variants. Important to understanding the etiology of mild RHUC (serum uric acid ≤ 3.0 mg/dL) is that it can be caused by a non-functional variant in heterozygotes. Other mutations such as the E298D mutation (rs121907894) and T217M mutation (rs121907893) have also been reported [15], but less frequently in the Japanese population.

Nakayama et al. [72] examined the low-risk allele fractional excretion of uric acid (FE_UA_) and the risk allele frequencies of non-functional URAT1 variants in 4993 Japanese people (R90H and W258X). Nine of the ten hypouricemic participants had non-functional variants and were evaluated as having RHUC. R90H and W258X have been proven to be significant risk alleles for hypouricemia. The only risk allele for patients with serum uric acid ≤ 3.0 mg/dL was W258X in the male population [18]. Based on epidemiological and genetic reports, RHUC should be suspected if the serum uric acid levels are 2.0 mg/dL or less. Moreover, in the case of men, there should be suspicion of RHUC at 3.0 mg/dL or less. Although RHUC is characterized by low serum uric acid levels and high FE_UA_, Kawamura et al., demonstrated that non-functional variants in URAT1 significantly increased FE_UA_ and decreased serum uric acid. FE_UA_ and serum uric acid levels can be estimated from the number of alleles of non-functional variants of URAT1 (W258X and R90H) [74].

Matsuo et al., investigated the health examination database of the Japan Maritime Self-Defense Force and performed mutational analysis of all the coding regions of the GLUT9 gene, identifying two distinct heterozygous missense mutations (R380W and R198C in GLUT9L, corresponding to R351W and R169C in GLUT9S). These GLUT9 mutations caused RHUC type 2 because of reduced urate reabsorption by renal proximal tubule cells [16,75,76]. The further identification of causative genes, including urate transporter genes, is awaited because there are cases of RHUC without mutations in either URAT1/*SLC22A12* or GLUT9/*SLC2A9*.

## 7. Xanthinuria

XDH/XOR catalyzes the conversion of hypoxanthine to xanthine and the conversion of xanthine to uric acid in the metabolic pathway of purine degradation, as well as reducing either NAD^+^ or O_2_. A human disease associated with genetically determined dysfunction of XOR is called xanthinuria due to the excretion of xanthine in urine. It was first reported in 1954 and was characterized by a marked decrease in urinary urate excretion (about 3–30 mg/day) and an increase in urinary xanthine excretion. Thus, xanthinuria is a disease of hypouricemia caused by loss of XDH/XOR. It is a rare disease, with only a few hundred cases reported so far, and can be distinguishable from other types of hypouricemia by its low urinary urate excretion. Xanthinuria is an autosomal recessive disorder of purine metabolism, characterized by severe hypouricemia (often with serum uric acid levels below 1 mg/dL) [77].

Xanthinuria is classified into two subtypes, type I and type II. Type I xanthinuria is associated with XDH/XOR deficiency, whereas type II xanthinuria involves XDH/XOR and aldehyde oxidase (a molybdoflavo enzyme similar to XDH/XOR). Most patients with xanthinuria type I or type II are asymptomatic, but some may develop urolithiasis, acute kidney injury, or myositis due to tissue deposition of xanthine. Classic xanthinuria can be easily identified by an allopurinol loading test [78]. Because allopurinol is converted to oxypurinol by XDH/XOR and aldehyde oxidase, allopurinol administration results in the detection of oxypurinol in the urine and serum of patients with xanthinuria type I, but not in those with type II [79].

Ichida et al., cloned human XDH/XOR [80] and showed that the XDH/XOR gene is the cause of classical xanthinuria type I [81]. Furthermore, they found that a functional defect in the human molybdenum cofactor sulfurase gene was the cause of classical xanthinuria type II; the human molybdenum cofactor sulfurase protein donates sulfur atoms to the molybdenum cofactor of XDH/XOR and aldehyde oxidase [82].

## 8. Fanconi Syndrome

Fanconi syndrome is a global dysfunction of the proximal tubule leading to excessive urinary excretion of amino acids, glucose, phosphate, bicarbonate, uric acid, and other solutes reabsorbed by the proximal tubule of kidneys. The clinical features of Fanconi syndrome are aminoaciduria, low molecular weight proteinuria, hypophosphatemia, metabolic acidosis, and glycosuria. The most severe complications are bone demineralization from urinary phosphate wasting and progressive decline in kidney function. Fanconi syndrome can be divided into congenital and acquired types. In children, congenital metabolic abnormalities are the most common cause of Fanconi syndrome, while in adults, drug abuse and diseases such as Sjögren’s syndrome, multiple myeloma, and lymphoma are the most common causes. The pathogenesis of Fanconi syndrome is unknown. However, most or all cases include widespread abnormality of proximal tubule carriers, leakage of brush border or basolateral cell membrane, inhibition or abnormal Na^+^- and K^+^-ATPase pumps, impaired mitochondrial energy production, or dysfunction of other organelles [83].

In 2010, it was reported that homozygous duplication of *SLC34A1*, encoding NaPi-IIa, causes autosomal recessive Fanconi’s syndrome with hypophosphatemic rickets and renal failure. This finding provides a human model of NaPi-IIa loss of function, constitutes evidence for the inclusion of NaPi-IIa in the list of proteins involved in human hypophosphatemic rickets and proximal tubulopathy, and validates the important role of the human NaPi-IIa co-transporter in renal phosphate processing and maintenance of whole-body phosphate homeostasis [84].

## 9. Drug-Induced Secondary Hypouricemia

Hypouricemia caused by RHUC or xanthinuria is rare, but drug-induced hypouricemia is relatively frequent, with cases identified in hospitalized patients with polypharmacy. In a report by Ogino et al. [45], the number of inpatients with hypouricemia, 31 out of 1220 (2.54%), was significantly higher than the number of outpatients with hypouricemia, which was 5 out of 3258 (0.2%) [33]. This transient presentation of drug-induced hypouricemia was considered to have negligible clinical significance. However, the Framingham study confirmed the J-curve phenomenon, in which low serum uric acid levels are associated with an increased risk of cardiovascular events [85]. The Syst-Euro study also reported that low serum uric acid levels in hypertensive patients whose blood pressure is controlled with calcium channel blockers are associated with increased cardiovascular events [86]. Because uric acid is the most potent oxidative stress scavenger among biological substances, a severe decrease in uric acid levels may cause cardiovascular events because of increased oxidative stress. This treatable condition can be reversed by eliminating the cause; however, care must be taken to ensure that it is not overlooked in daily practice.

The model of urate transport via a urate transporter is well known in the kidney, and this model can explain drug-induced hypouricemia. Transporters on the membrane are required for hydrophilic urate to pass through cells. In the proximal tubule, urate enters the cell across the luminal membrane via URAT1 for reabsorption, and urate moves across the basolateral membrane into the blood via GLUT9. URAT1 is targeted by many uricosuric agents such as probenecid, benzbromarone, and losartan. Conversely, several agents such as cisplatin, ifosfamide, tenofovir, sodium valproate, aminoglycoside antibiotics, and deferasirox have a toxic effect on the proximal tubule of kidneys and can cause renal Fanconi syndrome. In addition, the drug-induced syndrome of inappropriate antidiuresis hormone (SIADH) also results in hypouricemia, which is a common clinical problem. The agents associated with hypouricemia are mainly five classes: antidepressants, anticonvulsants, antipsychotic agents, cytotoxic agents, and pain agents [87]. Tanigchi et al. [88] reported that stimulation of the V1a receptor induces downregulation of GLUT9, together with upregulation of *ABCG2* and NPT1, which causes an increase in the clearance of uric acid in kidneys. This study revealed that hypouricemia seen in SIADH might be attributable to V1a receptor stimulation [88].

## 10. Hereditary RHUC

Genetic and environmental factors can change uric acid levels in the body. The estimates of the heritability of serum urate range from 30% to 70%. However, common genetic variation accounts for only 7.9% of the variation in serum urate levels [89]. Misawa et al., showed that the heritability described by the male and female *SLC22A12* variants exceeds 10%, indicating that rare variants underlie a significant portion of the “missing heritability” of serum urate levels [90]. Genetic studies have identified defects in the URAT1/*SLC22A12* and GLUT9/*SLC2A9* urate transporter genes as the causes of RHUC type 1 [15] and type 2 [16,75], respectively (Figure 1), although there are patients without dysfunctional mutations in these genes. Therefore, there may be unknown causative genes [38,39]. The non-functional variants in URAT1 of RHUC type 1 include W258X (rs121907892) and R90H (rs121907896). These have been reported to be the two most common causative variants in the Japanese population [69,89]. Ichida et al. [22] tested missense mutations (c.G269A, c.G412A, c.G490A, c.A1145T, and c.T1289C) and a 5 bp deletion (c.1639_1643del-GTCC) for urate transport activity by in vitro expression analysis in Xenopus oocytes. The mutation c.G774A, which is p.W258X in *SLC22A12*, was identified as the major *SLC22A12* RHUC allele (74.1%), and its c.G774A resulted in truncated immature hURAT1, and c.1639–1643 del-GTCCT was considered to reduce the routing of the hURAT1 protein to the apical membrane by disrupting the PDZ binding motif [91]. The frequencies of the W258X and R90H alleles in a total of 909 Korean subjects were 1.1% and 0.6%, respectively. The proportion of subjects with one or both mutant alleles was 3.2% (29/909 subjects) of all subjects, and it was 36.7% (11/30 subjects) in hypouricemia patients. The mutations in W258X and R90H are considered to constitute the predominant mutations in Japan and Korea [92]. Recent studies suggest that RHUC is not necessarily restricted to East Asian populations, as previously thought. Loss-of-function mutations affecting URAT1 have also been found in Caucasian populations in Macedonia, the United Kingdom, Spain, the Czech Republic, and Sri Lanka [93,94,95,96,97,98]. In addition, case reports of European patients with symptoms of RHUC have been published [99,100]. Therefore, limited awareness of RHUC, rather than a heterogeneous geographic distribution of it, may be the reason why causes outside of the Far East have not been found. According to Gabrikova et al., a survey of 881 Roma people randomly selected in Eastern Slovakia and the Czech Republic found the frequencies of minor alleles for L415_G417del and T467M to be 5.56% and 1.87%, respectively [101]. Currently, more than 150 patients with a loss-of-function mutation in the URAT1/*SLC22A12* gene have been found, most of them Asians. However, patients with RHUC type 1 have been reported in various ethnic groups and in countries that are not geographically adjacent [44].

## 11. Complications of Hypouricemia

RHUC is a common hereditary heterogeneous disorder characterized by impaired urate transport [102]. The incidence of RHUC has been reported to range from 0.12% to 0.72% [103]. Most RHUC cases are known to often be accompanied by urolithiasis and EIAKI, especially in subjects with hereditary hypouricemia [4,5,99]. The clinical signs of urolithiasis and EIAKI often lead to an early diagnosis of RHUC. Cases of EIAKI may require hemodialysis treatment but generally present with transient acute kidney injury. On the other hand, the excessive urinary excretion of urate often causes the formation of uric acid crystals, causing urolithiasis in subjects with hypouricemia. On the basis of genetic testing, Ichida et al., reported the prevalence of urolithiasis (8.5%) and EIAKI (6.5%) in subjects with hereditary RHUC [29]. However, this may have involved some bias because the study population was small (only 71 subjects) and all of the subjects were routinely evaluated at a university hospital. Thus, the study may have detected asymptomatic stones, which could have resulted in a higher prevalence of urolithiasis. Kuwabara et al., conducted a cross-sectional study to determine whether hypouricemia is a risk factor for diseases such as urolithiasis and EIAKI in the Japanese population, and after analyzing medical records, they concluded that hypouricemia per se does not increase urolithiasis in either men or women [56]. This result is interesting because it differs from previous reports that stated that urolithiasis is one of the complications of hypouricemia.

Uric acid is involved in a complex reaction with several oxidants and may have particular protective effects under certain conditions [48]. Because urate protects endothelial function as an antioxidant, hypouricemia causes EIAKI via renal artery spasms characterized by nausea, vomiting, back pain, abdominal pain, and general fatigue. Many cases of EIAKI in patients with RHUC have been reported [6,7,31]. There are two types of EIAKI. One is the well-known acute renal failure due to myoglobin, and the other is acute renal failure with severe back pain that develops after anaerobic exercise, which may not be due to myoglobin or may be induced by myolysis of type 2 muscle fibers due to anaerobic exercise. Ishikawa et al., reported acute renal failure with RHUC after anaerobic exercise, such as a 200 m track race in 1982. The patients complained of severe back pain several hours after exercise, but creatine phosphokinase and serum myoglobin levels were normal or only slightly elevated. Renal computed tomography scans performed hours to 1–2 days after contrast administration showed multiple wedge-shaped contrast enhancement areas. The pathogenesis of acute renal failure with severe back pain that develops after anaerobic exercise is thought to involve patchy vasoconstriction of the renal vessels, based on its wedge-shaped distribution and its reversibility. Such vascular spasms are thought to cause pain in the kidneys [4]. RHUC patients with homozygous or compound heterozygous mutations in URAT1 or GLUT9 should be advised to avoid structured exercise and to drink adequate fluids after exercise. Prevention with allopurinol and oral antioxidant supplementation is also recommended for preventing the recurrence of EIAKI episodes [104,105,106]. The purpose of using allopurinol in patients with hypouricemia is to reduce the production of uric acid, thus reducing the load of filtered uric acid and reducing the risk of uric acid precipitating into the tubules. Allopurinol, a XOR inhibitor, has been administered according to several case reports [105,106,107] for preventive purposes based on the hypothetical mechanism of EIAKI’s pathogenesis, but there is no convincing evidence of its efficacy. For urolithiasis, alkalizing urine with citrate compounds is an effective treatment [106], and it is advisable to drink plenty of fluids for prevention.

Although it may be a secondary change, the risk of cardiovascular events may also be increased in patients with hypouricemia [55,108,109,110]. Kuwabara et al., evaluated the number of Japanese subjects (and prevalence) in Japan over five years for each 1 mg/dL of serum urate stratified by gender. Low serum urate was an independent factor in predicting hypertension, dyslipidemia, and CKD. Furthermore, hypouricemia may cause an increased risk of developing hypertension and CKD in women [56].

## 12. Conclusions

Uric acid, despite being a major antioxidant in human plasma, correlates with the development of obesity, hypertension, and cardiovascular disease. Hyperuricemia is a known risk factor for gout; however, hypouricemia can go unnoticed because it is often asymptomatic, and complications such as post-exercise acute kidney injury in RHUC are not fully understood. We should inform physicians in their daily practice about the existence of hypouricemia. These physicians must recognize hypouricemia as a potentially harmful condition and should provide accurate diagnosis and treatment for patients. Therefore, herein, we have provided an updated review in the field of hypouricemia. We explained normal uric acid/urate metabolism and urate transporters and the risks associated with developing diseases caused by hypouricemia. We focused on urate transporters in the kidney, particularly URAT1/*SLC22A12*, a urate anion exchanger regulating serum uric acid levels, and described uricosuric agents and patients with renal hypouricemia who have mutational defects in *SLC22A12*. Recent studies have revealed the causative gene, and much knowledge has been gained about the pathogenesis of diseases associated with loss-of-function mutations. We expect to provide new information about uricosuric agents for other transporters and URAT1. We also expect readers to be interested in the paradoxical dynamics of uric acid as an oxidant–antioxidant.

## Figures and Tables

**Table 1:** Diseases that cause hypouricemia.

A. Overexcretion type hypouricemia
Renal hypouricemia (RHUC) (1) Type 1: URAT1/*SLC22A12* mutations (2) Type 2: GLUT9/*SLC2A9* mutations (3) Type 3: Unknown mutation type Fanconi syndrome Wilson’s disease Syndrome of inappropriate antidiuretic hormone secretion (SIADH) Malignancy Drugs (such as uricosuric agents)
B. Underproduction type hypouricemia
Xanthiuria (1) Type 1: XOR mutations (2) Type 2: MOCOS mutations Molybdenum cofactor deficiency (MOCOD) Purine nucleoside phosphorylase (PNP) deficiency Phosphoribosyl pyrophosphate (PRPP) synthetase deficiency Severe liver disorder Drugs (such as xanthine oxidase inhibitor) Malnutrition Idiopathic hypouricemia with underproduction of uric acid

## Data Availability

Not applicable.
